# Comparison of three multiplex PCR assays for the detection of respiratory viral infections: evaluation of xTAG respiratory virus panel fast assay, RespiFinder 19 assay and RespiFinder SMART 22 assay

**DOI:** 10.1186/1471-2334-12-163

**Published:** 2012-07-24

**Authors:** Mareike Dabisch-Ruthe, Tanja Vollmer, Ortwin Adams, Cornelius Knabbe, Jens Dreier

**Affiliations:** 1 Institut für Laboratoriums- und Transfusionsmedizin, Herz- und Diabeteszentrum Nordrhein-Westfalen, Universitätsklinik der Ruhr-Universität Bochum, Bad Oeynhausen, Germany; 2 Institut für Virologie, Universitätsklinikum, Düsseldorf, Germany

## Abstract

**Background:**

A broad spectrum of pathogens is causative for respiratory tract infections, but symptoms are mostly similar. Therefore, the identification of the causative viruses and bacteria is only feasible using multiplex PCR or several monoplex PCR tests in parallel.

**Methods:**

The analytical sensitivity of three multiplex PCR assays, RespiFinder-19, RespiFinder-SMART-22 and xTAG-Respiratory-Virus-Panel-Fast-Assay (RVP), were compared to monoplex real-time PCR with quantified standardized control material. All assays include the most common respiratory pathogens.

**Results:**

To compare the analytical sensitivity of the multiplex assays, samples were inoculated with 13 different quantified viruses in the range of 10^1^ to 10^5^ copies/ml. Concordant results were received for rhinovirus, whereas the RVP detected influenzavirus, RSV and hMPV more frequently in low concentrations. The RespiFinder-19 and the RespiFinder-SMART-22 showed a higher analytical sensitivity for adenoviruses and coronaviruses, whereas the RVP was incapable to detect adenovirus and coronavirus in concentrations of 10^4^ copies/ml. The RespiFinder-19 and RespiFinder-SMART-22A did not detect influenzaviruses (10^4^ copies/ml) and RSV (10^3^ copies/ml). The detection of all 13 viruses in one sample was only achieved using monoplex PCR. To analyze possible competitive amplification reactions between the different viruses, samples were further inoculated with only 4 different viruses in one sample. Compared to the detection of 13 viruses in parallel, only a few differences were found.

The incidence of respiratory viruses was compared in tracheal secretion (TS) samples (n = 100) of mechanically ventilated patients in winter (n = 50) and summer (n = 50). In winter, respiratory viruses were detected in 32 TS samples (64%) by RespiFinder-19, whereas the detection rate with RVP was only 22%. The most frequent viruses were adenovirus (32%) and PIV-2 (20%). Multiple infections were detected in 16 TS samples (32%) by RespiFinder-19. Fewer infections were found in summer (RespiFinder-19: 20%; RVP: 6%). All positive results were verified using monoplex PCR.

**Conclusions:**

Multiplex PCR tests have a broad spectrum of pathogens to test at a time. Analysis of multiple inoculated samples revealed a different focus of the detected virus types by the three assays. Analysis of clinical samples showed a high concordance of detected viruses by the RespiFinder-19 compared to monoplex tests.

## Background

Acute respiratory tract infections are the most widespread type of infection in adults and children and are responsible for a considerable morbidity and mortality worldwide. A high rate of respiratory tract infections is caused by viruses (approximately 80%) [1,2]. Within the last ten years, diagnosis of respiratory viruses has become more important, because of the unexpected emergence of several new respiratory viruses: (a) influenza A virus H5N1 (1997), (b) human metapneumovirus (hMPV; 2001; [1]), (c) coronavirus SARS (CoV-SARS; 2002); (d) mimivirus (2003; [2]); (e) coronavirus NL63 (CoV-NL63; 2004; [3]); (f) coronavirus HKU1 (CoV-HKU1; 2005; [4]), (g) human bocavirus (2005; [5]), (h) parvovirus 4 (2005; [6]), (i) influenza A virus H1N1 (INF-A; 2009). Due to the similarity in clinical presentation of respiratory tract infections, causative pathogens could not be identified on the basis of symptoms alone. However, an efficient pathogen-based prophylaxis or therapy has a significant effect on the disease progress in patients [7,8]. To overcome limitations concerning the use of several monoplex tests in parallel and the resulting shortage of sample volume, the development of multiplex tests for a fast and exact identification is necessary. Today, several multiplex tests are commercially available [9-16]. A number of studies have already compared the detection frequencies of multiplex assays with conventional monoplexPCR assays in clinical samples [12,17-19], but a comparison of the analytical sensitivity of these multiplex assays with quantified standardized control material does not exist.

This study presents the first comparison of the analytical sensitivity of three novel multiplex PCR methods, the RespiFinder-19 assay, RespiFinder-SMART(Single tube Multiplex Amplification in Real-Time)-22 assay (both PathoFinder, Maastricht, Netherlands) and the xTAG Respiratory Virus Panel Fast Assay (Abbott Molecular, Wiesbaden, Germany), with quantified virus control material. The RespiFinder-19 assay, which is based on the multiplex ligation-dependent probe amplification analyzed by capillary electrophoresis, detects 15 respiratory viruses and 4 bacteria in one reaction [20]. A further development of the RespiFinder-19 assay is the RespiFinder-SMART-22 assay, which is also based on the multiplex ligation-dependent probe amplification. This assay differentiates 18 respiratory viruses and 4 bacteria in one reaction by melt curve analysis. The xTAG Respiratory Virus Panel Fast Assay (RVP) is a bead array-based system for the detection of 19 different respiratory viruses [9,10]. Previous studies with clinical samples showed that the sensitivity and specificity of the RVP assay was 78.8% and 99.6%, respectively, compared to real-time PCR-methods, that are currently declared as the gold standard [21]. Another study performed a comparison with clinical samples of the RespiFinder-19 with the precursor assay of the RVP [7]. Both assays in this study have an excellent specificity and the sensitivity was 33% and 78% for the RVP and the RespiFinder-19 assay, respectively.

The aims of the present study were as follows: (a) the quantification of commercially available qualitative control material for (b) the evaluation of the performance and sensitivity of the three multiplex assays RespiFinder-19, RespiFinder-SMART-22 and RVP fast assay and (c) the applicability and performance of these multiplex PCR assays in a routine setting. Furthermore, this is the first screening study determining the incidence of infections with respiratory pathogens in a mechanically ventilated patient cohort developing an atypical pneumonia during post-operative monitoring using multiplex PCR assays.

## Methods

### Clinical specimen

Tracheal secretion (TS) samples (n = 100) were collected in February (winter, n = 50) and July (summer, n = 50) 2010 from mechanically ventilated non-immunocompromised patients (male: 59%, female: 41%, mean age 62.8 ± 23.2 years, range 23–91). Patients were suspected of atypical pneumonia during postoperative monitoring after coronary artery bypass, heart or lung surgery. TS samples were initially analyzed for bacterial and viral pathogens with our routine diagnostic profile including CMV, *Legionella pneumophila*, *Pneumocytisjirovecii*, *Mycoplasma pneumoniae* and *Chlamydophilapneumoniae*. The residual material was used for the comparative analysis with the RespiFinder-19 and RVP assay. The results of the performance evaluation were received retrospectively and had no influence on patient's therapy. All patients provided informed consent. For our study we did not need an ethical approval, because in paragraph §24 of the German Act on Medical Devices (in German: GesetzueberMedizinprodukte - MPG) an ethical approval is not required for clinical specimens without a separate invasive sampling.

### Virus controls

The qualitative Respiratory Validation Panel Global (NATRVP-2, ZeptoMetrix Corporation, Buffalo, New York) was quantified by real-time qPCR. External plasmid standards were used to determine the concentration of the different viruses. Plasmid standards of adenovirus (from position 18895 to 18968, accession number AC_000008), influenza A virus (from position 144 to 238, accession number CY041531), RSV-A (from position 1801 to 1949, accession number M11486), enterovirus (from position 455 to 602, accession number D00820) and rhinovirus (from position 356 to 563, accession number D00239) were established [15]. Samples were analyzed in triplicate in three independent PCR assays. The viruses hMPV, PIV-1, PIV-2 and PIV-3 were quantified by LightMix assays (TIB MOLBIOL, Berlin, Germany).

The virus controls of the NATRVP-2 are purified intact virus particles that have been chemically modified to render them non-infectious and refrigerator stable. Quantifications of viruses included in this panel were as follows: influenza A virus (H1: 2.31E + 05 copies/ml, H3: 1.85E + 05 copies/ml), RSV-A (5.12E + 05 copies/ml), adenovirus (4.63E + 05 copies/ml), rhinovirus (3.68E + 05 copies/ml), hMPV (2.31E + 05 copies/ml), PIV-1 (2.09E + 05 copies/ml), PIV-2 (1.19E + 05 copies/ml) and PIV-3 (4.99E + 05 copies/ml) (Table 1).

**Table 1:** Key parameters for monoplex real-time PCR assays

**Virus**	**Dynamic range**	**Efficiency**	**Interassay- variabilty**	**R2**
INF-A H1	1.00E + 05 - 1.00E + 01	1.89	22.7 ± 0.49	0.972
INF-A H3	1.00E + 05 - 1.00E + 01	1.89	22.7 ± 0.49	0.972
RSV-A	1.00E + 06 - 1.00E + 01	1.98	22.4 ± 0.36	0.998
PIV-1	1.00E + 06 - 1.00E + 01	1.91	29.9 ± 0.84	0.993
PIV-2	1.00E + 05 - 1.00E + 01	1.95	29.3 ± 0.33	0.997
PIV-3	1.00E + 06 - 1.00E + 01	1.88	36.4 ± 0.30	0.989
HRV	1.00E + 06 - 1.00E + 01	1.90	21.3 ± 0.34	0.991
AdV	1.00E + 06 - 1.00E + 01	1.88	19.9 ± 0.17	0.989
hMPV	1.00E + 06 - 1.00E + 01	1.87	26.7 ± 0.66	0.986

*Concentration of viruses are concordant to Table 2.

For the undiluted samples with 13 viruses, 22 μl of each virus were mixed (total volume: 286 μl). Accordingly, for the undiluted samples inoculated with 4 viruses, 22 μl of each virus were mixed and filled up with PBS to achieve also a final volume of 286 μl. Subsequently, 200 μl of the sample was extracted using the NucliSENSeasyMAG automated system with an elution volume of 55 μl.

### Nucleic acid extraction

The total nucleic acid from inoculated samples and tracheal secretion samples (TS) was extracted using the NucliSENSeasyMAG automated system (bioMérieux, Nürtingen, Germany) according to the manufacturer’s instructions. Nucleic acids were extracted from 500 μl of TS samples and 200 μl of inoculated samples and were eluted in a final volume of 55 μl (routine diagnostic) or 100 μl elution buffer, respectively (bioMérieux, Nürtingen, Germany). The manufacturer of the Respi-Finder-19 and SMART-22 assays (Pathofinder) recommend an elution volume of 100 μl, whereas the manufacturer of the RVP assay recommend an elution volume of 55 μl. To avoid false negative results due to a deviation from the manufacturer instructions, we extracted residual material and used an elution volume of 100 μl.

### xTAG Respiratory Virus Panel Fast Assay (RVP)

The nucleic acid extracts were tested using the RVP assay (Abbott Molecular, Wiesbaden, Germany) according to the manufacturer’s instructions. The RVP employs a multiplex PCR with labelled primers and a single-step hybridization of PCR products to the fluorescent bead array. The detection was performed using the xMAP 100 IS instrument (Luminex Molecular Diagnostics Inc., Toronto, Canada) and the analysis was performed using TDAS RVP FAST software (version 2.0, Abbott Molecular).

The RVP simultaneously detects influenza A virus (subtyped as H1, H3 or H5), influenza B virus, RSV-A and -B, adenovirus, hMPV, PIV-1, -2, -3 and −4, coronaviruses 229E, NL63, OC43 and HKU1, picornavirus (enterovirus and rhinovirus) and human bocavirus. The assay also includes an internal positive control added to each specimen at the extraction step (phage MS2) and a positive run control that is added to each plate (phage lambda DNA) [16].

### RespiFinder-19 and RespiFinder-SMART-22 assay

The RespiFinder-19 and the RespiFinder-SMART-22 (both PathoFinder, Maastricht, The Netherlands) were used according to the manufacturer’s instructions except for the nucleic acid extraction of patient samples, because we use 500 μl of TS samples instead of 200 μl as recommend. Elution volumes were used as described previously. Briefly, the assays comprised a preamplification step, which combines reverse transcriptase and multiplex target amplification PCR, followed by a probe hybridization step, a probe ligation step and a probe amplification step. The RespiFinder-19 analyzes the amplified PCR products by capillary electrophoresis using a DNA analyzer (ABI 3730, Applied Biosystems, Darmstadt, Germany), whereas the RespiFinder-SMART-22 analyzes by melt curve analysis on the RotorGene Q (Qiagen, Hilden, Germany).

The RespiFinder-19 simultaneously detects 15 respiratory viruses (adenovirus, coronaviruses 229E, NL63, OC43, hMPV, influenza A virus, influenza A virus H5N1, influenza B virus, PIV-1, -2, -3 and −4, RSV-A and -B, rhinovirus) and four bacteria (*Bordetella pertussis*, *Chlamydophila pneumoniae*, *Legionella pneumophila*, *Mycoplasma pneumoniae*). The RespiFinder-SMART-22 additionally detects coronavirus HKU1, enterovirus and bocavirus. Both assays also tests an internal positive control added to each specimen at the extraction stage (RNA transcript of the polyprotein gene from encephalomyocarditis virus) [20].

### Monoplex real-time PCR

PCR primers and probes were adapted as previously described: adenovirus [17], coronavirus (types NL63, HKU-1, OC43, 229E) [18], cytomegalovirus (CMV) [19], enterovirus [22], influenza A virus [23], RSV-A [22], rhinovirus [24] and *Legionella pneumophila*[25]. The bacteriophage MS2 was used as an internal control (IC) for the reverse transcription PCR as previously described [26]. A 89 bp fragment of the bacteriophage lambda (position 2402 to 2491, accession number J02459) was used as IC to avoid competitive co-amplification of DNA viruses.

RNA amplification of coronavirus (types NL63, HKU-1, OC43, 229E), influenza A virus, RSV-A and rhinovirus was carried out in 0.2 ml tubes containing 45 μl reaction mix and 5 μl RNA extract. The reaction mix consisted of 2 × Invitrogen rnx-reaction mix (including 50 mM MgSO_4_), 400 nM of target primers, 100 nM of the target probe, 200 nM of IC primers, 100 nM of the IC probe and 5 U of Invitrogen SuperScript Platinum *Taq*-enzym mix (SuperScript III One-Step RT-PCR with Platinum-Taq, Invitrogen, Darmstadt, Germany). A 203-bp PCR product of the bacteriophage MS2 replicase gene was added to the reaction mixture as an exogenous IC. PCR was performed on the RotorGene Q system (Qiagen, Hilden, Germany) with a reverse transcription at 50°C for 15 min, preliminary denaturation at 95°C for 10 min, followed by 45 cycles of denaturation of 95°C for 15 s, annealing and extension at 60°C for 45 s, with a single fluorescence acquisition step at the end of the annealing step.

DNA amplification of CMV, adenovirus and enterovirus was carried out in 0.2 ml tubes containing 45 μl reaction mix and 5 μl DNA extract. The reaction mix consisted of 10 × Taq buffer (including 50 mM Mg), 400 nM of each deoxynucleoside triphosphate, 500 nM of target primers, 200 nM of the target probe, 300 nM of IC primers, 100 nM of the IC probe, 0.01 U of Uracil-DNA Glycosylase (UNG, Roche Diagnostics, Mannheim, Germany) and 5 U of *Taq* DNA polymerase (5 PRIME, Hamburg, Germany). PCR was performed on the Rotor-Gene 3000 system (Corbett Life Sciences, Sydney, Australia) with an UNG activity step at 37°C for 5 min, preliminary denaturation at 95°C for 10 min, followed by 45 cycles of denaturation of 95°C for 15 s, annealing and extension at 65°C for 45 s, with a single fluorescence acquisition step at the end of the annealing step.

The *Legionella pneumophila* PCR was carried out in glass capillaries containing 15 μl reaction mix and 5 μl DNA extract. The reaction mix consisted of 5 × FastStart DNA Master Plus Hybridization Probes (Roche Diagnostics, Mannheim, Germany), 200 nM of each target primer, 150 nM of the target probe, 300 nM of each IC primer for lambda, 100 nM of the IC probe and 0.01 U of UNG (Roche Diagnostics, Mannheim, Germany). PCR was performed on the LightCycler 2.0 system (Roche Diagnostics, Mannheim, Germany) with an UNG activity step at 37°C for 10 min, preliminary denaturation at 95°C for 10 min, followed by 45 cycles of denaturation of 95°C for 2 s, annealing at 55°C for 10 s and extension at 72°C for 15 s, with a single fluorescence acquisition step at the end of the annealing step, and a following melt analysis (95°C for 60 s and 40°C for 30 s).

The real-time PCR for hMPV, PIV-1, -2 and −3 was performed using LightMix assays (TIB MOLBIOL, Berlin, Germany) on the LightCycler 2.0 system according to the manufacturer’s instructions.

The cut-off value for the decision positive/negative was adjusted to <40 cycles. The specificity of all monoplex-real-time PCR assays was determined by the exclusion of cross-amplification with different bacterial or viral DNAs/RNAs (eight bacteria, seven viruses). The analytical sensitivity was determined to be <10 copies/ml. The reproducibility of the assay was demonstrated by analyzing the inter-assay variation for the crossing threshold (*CT*) values, determined from six independent PCR runs. Key parameters for real-time PCR assays are shown in Table 1.

## Results

### Comparison of xTAG Respiratory Virus Panel Fast assay (RVP) with RespiFinder-19 assay and RespiFinder-SMART-22 assay using virus control material

Virus control material, NATRVP2 (Zeptometrix), was quantified by external plasmid standards for a comparison of the analytical sensitivity of the RVP, RespiFinder-19 and RespiFinder-SMART-22 assays. Subsequently, PBS-buffer was inoculated with 13 different quantified viruses, or 4 viruses in different combinations, in the range of 10^1^-10^4^ copies/ml. Nucleic acid extracts were analyzed in parallel with the three multiplex methods and monoplex real-time PCR methods (elution volume 55 μl). The RVP showed the detection of influenzavirus A (INF-A, 1.78E + 02 copies/ml), respiratory syntical virus A and B (RSV-A/B, 3.94E + 02 copies/ml), coronavirus OC43 (CoV OC43) and human metapneumovirus (hMPV, 1.78E + 01 copies/ml) even in a high dilution ratio (Table 2). Rhinovirus (HRV) was also detected in low concentration by RVP; however this assay is not able to differentiate between human rhinovirus and enterovirus due to the high sequence similarity (manufacturer’s information). INF-B, CoV 229E, parainfluenzavirus 1–3 (PIV-1-3) and adenovirus (AdV) were not detected. In contrast, the RespiFinder-19 and the RespiFinder-SMART-22 detected AdV and CoV OC43 and 229E also at low concentrations (e.g. AdV 3.56E + 02). However, both assay did not detect INF-A or -B, RSV-A or B, PIV-1 and −3 as well as hMPV in concentrations in the range of 10^4^ copies/ml. Only the RespiFinder-SMART-22 detected PIV-2 at a concentration of 92 copies/ml). None of the three multiplex assays was capable of detecting all 13 viruses in parallel. This was only achieved using monoplex real-time PCR assay. CT values were shown in Table 2.

**Table 2:** Comparison of RVP, RespiFinder-19, RespiFinder-SMART-22 and monoplex real-time PCR with regard to the analytical sensitivity in a sample with 13 viruses

**Virus**	**Concentration [copies/ml]**	**RVP**				**RespiFinder-19**				**RespiFinder- SMART-22**				**Monoplex real-time PCR**			
		**und.**	**1:10**	**1:100**	**1:1000**	**und.**	**1:10**	**1:100**	**1:1000**	**und.**	**1:10**	**1:100**	**1:1000**	**und.**	**1:10**	**1:100**	**1:1000**
INF-A H1	1.78E+04	**+**	**+**	**+**	**-**	**-**	**-**	**-**	**-**	**-**	**-**	**-**	**-**	32.9	33.8	35.8	37.9
INF-A H3	1.42E+04	**+**	**+**	**+**	**-**	**-**	**-**	**-**	**-**	**-**	**-**	**-**	**-**				
INF-B	n. q.	**-**	**-**	**-**	**-**	**-**	**-**	**-**	**-**	**-**	**-**	**-**	**-**	x	x	x	x
RSV-A	3.94E+04	**+**	**+**	**+**	**-**	**-**	**-**	**-**	**-**	**-**	**-**	**-**	**-**	28.7	30.4	32.5	34.6
RSV-B	n. q.	**+**	**+**	**+**	**-**	**-**	**-**	**-**	**-**	**-**	**-**	**-**	**-**	x	x	x	x
PIV-1	1.61E+04	**-**	**-**	**-**	**-**	**-**	**-**	**-**	**-**	**-**	**-**	**-**	**-**	32.8	33.1	34.9	36.6
PIV-2	9.19E+03	**-**	**-**	**-**	**-**	**+**	**-**	**-**	**-**	**+**	**+**	**+**	**-**	29.5	31.4	33.7	36.4
PIV-3	3.84E+04	**-**	**-**	**-**	**-**	**-**	**-**	**-**	**-**	**-**	**-**	**-**	**-**	34.9	36.8	38.3	39.8
CoV OC43	n. q.	**+**	**+**	**+**	**+**	**+**	**+**	**+**	**+**	**+**	**+**	**+**	**+**	27.6	28.9	30.1	31.9
CoV 229E	n. q.	**-**	**-**	**-**	**-**	**+**	**+**	**+**	**+**	**+**	**+**	**+**	**+**	26.8	27.9	29.1	30.5
HRV	2.83E+04	**+**	**+**	**+**	**+**	**+**	**+**	**-**	**-**	**+**	**+**	**+**	**-**	31.2	33.8	36.0	38.4
AdV	3.56E+04	**-**	**-**	**-**	**-**	**+**	**+**	**+**	**+**	**+**	**+**	**+**	**-**	35.9	37.6	38.8	39.9
hMPV	1.78E+04	**+**	**+**	**+**	**+**	**-**	**-**	**-**	**-**	**-**	**-**	**-**	**-**	30.2	32.0	34.8	38.4

n. q.: not quantified; x: not performed; und.: undiluted; The dilution series were done as two-fold dilution series.

In order to simulate clinical-relevant multiple infections as well as to analyse possible competitive amplification reactions between the different viruses, PBS-buffer was further inoculated with only 4 different viruses in one assay (Table 3). The nucleic acid extraction was done with two different elution volumes, 55 μl (according to Abbott Molecular instructions) and 100 μl (according to PathoFinder instructions), to avoid false negative results due to a deviation from the manufacturer instructions. We tested different viruses in three combinations (Panel 1: INF-A H1N1, RSV-A, HRV and AdV; Panel 2: INF-A H1N1, INF-A H3N2, RSV-A, PIV-2; Panel 3: CoV OC43, CoV 229E, AdV and HRV). Compared to the detection of 13 viruses in parallel, only a few differences were found using the same elution volume of 55 μL. RSV-A and AdV were now detected by the RespiFinder-19 and RespiFinder-SMART-22 assays at concentrations ranging from 10^4^ copies/ml (panel 1) to 10^2^ copies/ml (RSV panel 2, AdV panel 3). The RVP assay now detect AdV (10^4^ copies/ml panel 1, 10^3^ copies/ml panel 3) and PIV-2 (10^4^ copies/ml, panel 2). The RVP was still incapable of detecting CoV 229E. However, more differences were found in the detection of the pathogens using different elution volumes for both RespiFinder assays. The company PathoFinder suggests an elution volume of 100 μl for the RespiFinder-19 and the RespiFinder-SMART-22 assay, whereas Abbott Molecular advises for the RVP assay an elution volume of 55 μl. The two assays of the company PathoFinder did not detect INF-A in samples which were extracted in 55 μl elution buffer, but detection was possible by using the advised extraction volume of 100 μl. The RespiFinder-19 detect INF-A at a concentration of 10^4^ copies/ml (panel 1), whereas both assays detect INF-A at concentrations ranging from 10^2^ -10^4^ copies/ml (panel 2). The same observation was made for the detection of AdV at a concentration of 10^2^ copies/ml (panel 1) with the RespiFinderSMART-22 by using the advised extraction volume of 100 μl. In contrast, the detection frequency of RSV-A virus was reduced for both RespiFinder assays analysing samples with 100 μl elution volume compared to 55 μl elution volume (panel 1: undiluted sample; panel 2: RespiFinder19 all samples, RespiFinderSMART-22 dilution 1:100). No differences in the detection frequency were found for the RVP assay (Table 3).

**Table 3:** Comparison of RVP, RespiFinder-19, RespiFinder-SMART-22 and monoplex real-time PCR with regard to the analytical sensitivity in a sample inoculated with 4 viruses

	**RVP**			**RespiFinder-19**			**RespiFinder-SMART-22**			**Monoplex real-time PCR (CT)**		
	**und.**	**1:10**	**1:100**	**und.**	**1:10**	**1:100**	**und.**	**1:10**	**1:100**	**und.**	**1:10**	**1:100**
Panel 1	55 μl elution			55 μl elution			55 μl elution			55 μl elution		
(INF-A H1/ RSV-A/ HRV/ AdV)	**+ / + / + / +**	**+ / + / + / -**	**+ / - / + / -**	**- / + / + / +**	**- / - / + / -**	**- / - / - / -**	**- / + / + / +**	**- / - / + / -**	**- / - / + / -**	21.8/22.3/ 20.4/28.7	25.8/26.9/ 26.8/30.5	29.6/28.3/ 30.8/31.7
	100 μl elution			100 μl elution			100 μl elution					
	**+ / + / + / +**	**+ / + / + / -**	**+ / - / + / -**	**+ / - / + / +**	**+ / - / + / -**	**- / - / - / -**	**- / - / + / +**	**- / - / + / +**	**- / - / + / +**			
Panel 2*	55 μl elution			55 μl elution			55 μl elution			55 μl elution		
(INF-A H1/ INF-A H3/ RSV-A/ PIV-2)	**+ / + / + / +**	**+ / + / + / -**	**+ / + / - / -**	**- / - / + / +**	**- / - / + / +**	**- / - / + / +**	**- / - / + / +**	**- / - / + / +**	**- / - / + / +**	24.3/24.3/ 22.8/20.4	28.7/28.7/ 25.5/26.4	31.1/31.1/ 28.7/30.4
	100 μl elution			100 μl elution			100 μl elution					
	**+ / + / + / +**	**+ / + / + / -**	**+ / + / - / -**	**+ / + / - / +**	**+ / + / - / +**	**+ / + / - / -**	**+ / + / + / +**	**+ / + / + / +**	**+ / + / - / +**			
Panel 3*	55 μl elution			55 μl elution			55 μl elution			55 μl elution		
(CoV OC43/ CoV 229E/ AdV/ HRV)	**+ / - / + / +**	**+ / - / + / +**	**- / - / - / +**	**+ / + / + / +**	**+ / + / + / +**	**+ / + / + / -**	**+ / + / + / +**	**+ / + / + / +**	**+ / + / + / +**	25.0/22.3/ 29.7/22.4	28.7/25.3/ 31.8/25.4	32.0/26.5/ 32.6/28.5
	100 μl elution			100 μl elution			100 μl elution					
	**+ / - / + / +**	**+ / - / + / +**	**- / - / - / +**	**+ / + / + / +**	**+ / + / + / +**	**+ / + / + / -**	**+ / + / + / +**	**+ / + / + / +**	**+ / + / + / +**			

*Concentration of viruses are concordant to Table 1.

### Detection of respiratory viruses in clinical samples

Additionally, we evaluated the applicability of the RVP and the RespiFinder-19 for routine diagnosis and the distribution of viruses in our patient cohort in 100 TS samples during winter (February) and summer (July). The RespiFinder-SMART-22 as a further development of the RespiFinder-19 was not participating in this study, because it started before the RespiFinder-SMART-22 was commercially available. All positive results were verified with real-time PCR as monoplex analysis. TS samples were obtained routinely from mechanically ventilated patients after coronary artery bypass, heart or lung surgery who were suspected of atypical pneumonia during post-operative monitoring.

**Figure 1:**
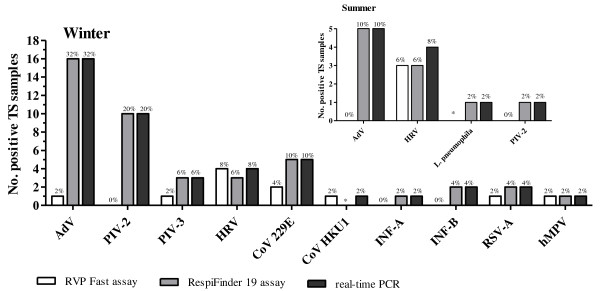
**Comparison of RVP, RespiFinder-19, and real-time PCR results for TS samples in winter and summer.** The rectangular boxes symbolize the different assays. * *L. pneumophila* is not in the spectrum of pathogens of the RVP and CoV HKU1 is not in the spectrum of the RespiFinder-19.

Respiratory viruses were detected by RespiFinder-19 in 32 TS samples from winter (64%) (Figure 1). The most frequent viruses were AdV, which was found in 16 TS samples (32%), and PIV-2, which was found in 10 TS samples (20%). Other detected viruses were CoV 229E (10%), PIV-3 (6%), HRV (6%), INF-B (4%), RSV-A (4%), INF-A (2%) and hMPV (2%). In comparison, the RVP detected respiratory viruses only in 11 TS samples (22%). Rhinovirus, which was found in 4 TS samples (8%), was the most frequent virus in addition to CoV 229E (4%), CoV HKU1 (2%), AdV (2%), PIV-3 (2%), RSV-A (2%) and hMPV (2%). In summer fewer respiratory virus infections were found in the TS samples by the different assays. The RespiFinder-19 detected in 10 TS samples (20%) respiratory viruses in contrast to the RVP, which found viruses only in 3 TS samples (6%), respectively (Figure 1). Furthermore, the spectrum of detected viruses was smaller in summer: AdV (10%), HRV (8%) and PIV-2 (both 2%). Additionally, *L. pneumophila* was detected by the RespiFinder-19 (2%). In order to confirm positive results obtained by RVP and RespiFinder, samples were additionally tested by monoplex real-time PCR with 100% concordance (Figure 1).

The analyses of three TS samples with RVP failed (Table 4). The error message “Sample failure in saline due to unexpected control call” appeared. The manufacturer provided the information that this is possible, if the sample is positive for a pathogen which is not in the spectrum of pathogens of the kit. In these three cases the samples contained CMV (detection only with monoplex real-time). In order to exclude CMV pneumonia due to reactivation processes, samples had already been tested for CMV in line with our standard diagnostic profile, and data was provided for completeness.

**Table 4:** Patient samples with positive pathogen detection from winter and summer

**Patient**	**Sex**	**Age (yr)**	**RVP**	**RespiFinder-19**	**real-time PCR**
*winter*					
*triple infections*					
1	m	71	-	INF-B, PIV-2	INF-B, PIV-2, CMV
*double infections*					
2	m	52	RSV	RSV-A, CoV 229E	RSV-A, CoV 229E
3	m	39	CoV 229E	CoV 229E	CoV 229E, CMV
4	f	84	CoV HKU1	CoV 229E, AdV	CoV HKU1, AdV
5	m	59	-	PIV-2	PIV-2, CMV
6	f	84	x	AdV	AdV, CMV
7	m	56	-	PIV-2	PIV-2, CMV
8	m	61	-	PIV-2, AdV	PIV-2, AdV
9	f	23	-	PIV-2, AdV	PIV-2, AdV
10	f	84	-	AdV	AdV, CMV
11	f	83	-	PIV-2, RSV-A	PIV-2, RSV-A
12	m	85	-	PIV-2, AdV	PIV-2, AdV
13	m	79	-	AdV, PIV-3	AdV, PIV-3
14	f	76	-	PIV-2, AdV	PIV-2, AdV
15	m	56	-	AdV, PIV-3	AdV, PIV-3
16	m	71	x	AdV	AdV, CMV
17	m	74	-	INF-A, PIV-2	INF-A, PIV-2
*mono infections*					
18	f	84	AdV	AdV	AdV
19	f	80	hMPV	hMPV	hMPV
20	m	65	PIV-3	PIV-3	PIV-3
21	m	73	-	AdV	AdV
22	m	70	-	AdV	AdV
23	m	79	-	PIV-2	PIV-2
24	f	62	-	AdV	AdV
25	f	72	HRV	HRV	HRV
26	f	82	HRV	HRV	HRV
27	m	80	-	AdV	AdV
28	m	71	CoV 229E	CoV 229E	n. d.
29	m	64	-	AdV	AdV
30	m	58	-	AdV	AdV
31	f	46	HRV	-	HRV
32	f	75	HRV	HRV	HRV
33	m	52	-	INF-B	INF-B
*summer*					
*double infections*					
34	f	74	HRV	HRV	HRV, CMV
35	m	70	x	AdV	AdV, CMV
*mono infections*					
36	m	59	HRV	-	HRV
37	f	77	-	-	CMV
38	f	78	-	-	CMV
39	m	81	-	-	CMV
40	f	43	-	HRV	HRV
41	m	73	-	AdV	AdV
42	f	91	HRV	HRV	HRV
43	m	66	-	PIV-2	PIV-2
44	m	59	-	AdV	AdV
45	f	65	-	AdV	AdV
46	f	48	-	*L. pneumophila*	*L. pneumophila*
47	m	66	-	AdV	AdV

f: female; m: male; x: sample failure in Saline due to unexpected control call.

In 16 TS samples (32%) from February, multiple infections were detected (Table 4). One triple infection with INF-B, PIV-2 and CMV was found. Double infections with adenovirus and PIV-2 (8%) as well as adenovirus and CMV (6%) were the most frequent combination. In July, double infections were only detected in 2 TS samples (4%). The detection of multiple infections was only possible with RespiFinder-19. The highest detection rate of 43 viruses in all samples was reached with the RespiFinder-19. A confirmation reaction was performed with monoplex PCR assay, which found a total of 45 viruses in 100 TS samples. The viruses which were only detected with RespiFinder-19 showed high cycle threshold points in monoplex PCR methods due to low virus concentrations.

## Discussion

The detection of respiratory viruses by multiplex PCR has been described as an important tool for the identification of the pathogens in respiratory tract infections.

But until today no comparison of these three multiplex PCR methods with quantified standardized virus control material was performed. Therefore, we quantified virus control material to compare the analytical sensitivity of three commercially available multiplex PCR methods, followed by evaluation of application for routine diagnosis with regard to hands-on-time, time-to-result, costs and accomplishment (Table 5).

**Table 5:** Comparison of RVP, RespiFinder-19 and RespiFinder-SMART-22 with regard to test specifications, performance, cost and accomplishment

	**RVP**	**RespiFinder-19**	**RespiFinder-SMART-22**
**Manufacturer**	Abbott Molecular	PathoFinder	PathoFinder
**CE IVD/FDA**	yes/yes	yes/no	yes/no
**TEST SPECIFICATIONS**			
**Pathogens**	influenza A + B	influenza A + B	influenza A + B
	RSV-A + B	RSV-A + B	RSV-A + B
	adenovirus	adenovirus	adenovirus
	hMPV	hMPV	hMPV
	PIV-1, -2, -3, -4	PIV-1, -2, -3, -4	PIV-1, -2, -3, -4
	CoV 229E, NL63, OC43, HKU1	CoV 229E, NL63, OC43	CoV 229E, NL63, OC43, HKU1
	HRV	HRV	HRV
	enterovirus	-	enterovirus
	bocavirus	-	bocavirus
	-	*B. pertussis*	*B. pertussis*
	-	*C. pneumoniae*	*C. pneumoniae*
	--	*L. pneumophilaM. pneumoniae*	*L. pneumophilaM. pneumoniae*
**Sample volume**	500 μL	200 μL	200 μL
**Elution volume**	55 μL	100 μL	100 μL
**PCR volume**	10 μL	10 μL	10 μL
**Principle of detection**	fluorescence bead array	capillary electrophoresis	melt curve analysis
**PERFORMANCE**			
**Hands-on-time**	0.80 h	1.75 h	1.25 h
**Time-to-result**	3.5 h	7.5 h	6.0 h
**COSTS**			
Costs reagents *^,1^	90-100 €	50-60 €	50-60 €
Costs equipment *	57-69,000 €	100-120,000 €	33-40,000 €
**ACCOMPLISHMENT**			
**Handling**	+	+	++
**Personal training duration/qualification**	2 d/very low	2 d/very low^2^	2 d/low
**Technical equipment**	extraction system, thermocycler, Luminex 200	extraction system, thermocycler, DNA-Sequencer ABI3500	extraction system, thermocycler, Rotorgene Q

*All prices are for Germany (list price).

^1^Prices for one sample.

^2^The personal training will be very low, if technicians are familiar with sequencing using an ABI system.

We observed that the RVP, the RespiFinder-19 and the RespiFinder-SMART-22 assay had a different focus of the detected virus types in the inoculated samples. The RVP showed an advanced detection of INF-A, RSV, and hMPV, whereas the RespiFinder-19 and the RespiFinder-SMART-22 showed an improved detection of CoV 229E and AdV. The RespiFinder-SMART-22 further showed an improved detection of PIV-2. Viruses were detected also at low concentrations of 10^1^-10^2^ copies/ml. CoV OC43 and HRV were detected equally also at low concentrations with the three assays, whereas PIV-1 and PIV-3 were not detected at all. The parallel analysis with monoplex real-time PCR assays showed as expected the highest analytical sensitivity: all viruses were detected in all concentrations and dilution factors.

Our results indicated a possible competition for nucleotides, primer or enzymes between the different viruses in the detection of a high number of multiple infections (13 viruses in one assay). 13 viruses in one sample was the maximum demand on the multiplex PCR tests. This highly artificial experiment emphasizes the general methodological limitation of multiplex PCR assay, because nearly all multiplex assays performed terribly analyzing samples with a high number of parallel pathogens. Therefore, these results have to be interpreted with attention. In the routine clinical setting, a parallel infection with this high number of different viruses is uncommon up to impossible, and the clinical relevance of these results is initially arguable. The highest virus load in our patient cohort was a triple infection. This was also observed by other research groups [8]. Due to this fact we tested three different combinations of quadruple infections. Surprisingly, only a few differences were found compared to the highly artificial experiment with 13 viruses. Potential competitive reactions were found for the detection of RSV and AdV (RespiFinder) or INF-A, AdV and PIV-2 (RVP). However, some viruses were still not detected (e.g. RVP: CoV 229E, RespiFinder: INF-A). But we observed an influence of the elution volumes for the detection with the RespiFinder-19 and the RespiFinder-SMART-22. An elution volume of 100 μl showed the tendency of a higher analytical sensitivity. However, the elution volume seems to have differing influences depending on the contained viruses. For example, INF-A was detected more frequent in an elution volume of 100 μl, whereas RSV showed a higher detection frequency using an elution volume of 55 μl. No differences were found in the detection with the RVP assay, although we expected that the dilution of the DNA/RNA extract may result in a reduced analytical sensitivity. For routine testing using this different multiplex assays in parallel, we suggest an elution volume of 100 μl, because the remaining extract is available for retesting or analysis for non-included pathogens. If only one multiplex assay will be implemented, elution should be performed according to the specifications of the manufacturer.

The analysis of 100 TS samples was done in parallel with the RVP and the RespiFinder-19. At that time, the RespiFinder-SMART-22 was not commercially available and later on, no residual extracted material was available for additional testing. For a comparison of the three multiplex assays with clinical samples further studies have to be done. This study indicated a higher clinical sensitivity of the RespiFinder-19 in the detection of virus infections in clinical samples with low concentrations in contrast to the RVP. Respiratory tract infections were found in 64% of the TS samples by RespiFinder-19. The RVP detected only 22% in the same samples, respectively. Gadsby *et al.*[21] also described problems in the detection of low virus concentrations with RVP; either the pathogens were not detected or they produced a false-positive for adenovirus. Raymaekers *et al.*[7] investigated clinical samples for a comparison of the RespiFinder-19 with the precursor assay of the RVP (xTAG Respiratory Viral Panel Assay, Abbott Molecular, Wiesbaden, Germany). The precursor assay of the RVP included a target specific primer extension (TSPE) in contrast to the new assay, therefore these results are not directly comparable to the present study. In Raymaekers’ study, respiratory viruses were detected by RVP in 31 of 95 clinical samples and by RespiFinder-19 in 75 of 95. These results are comparable to our results and verified our results in present study. A comparison of the precursor assay of the RVP with the newer RVP assay was made by Pabbaraju *et al.*[9]. They showed that the older assay was more sensitive than the newer RVP Fast assay (88.6% and 77.5% sensitivities, respectively) for all the viral targets combined. This corresponds to our observations concerning the RespiFinder-19. However, the higher sensitivity of the RespiFinder-19 assay may also be due to the detection of incidental but non-causal viruses. In 32% of the February TS samples the RespiFinder-19 assay detected multiple infections, corresponding to results of Fox [10], whereas the RVP assay detected no multiple infections. Raymaekers *et al.* also described problems in the detection of co-infections with the RVP. Samples which include adenovirus and coronavirus were false-negative with the RVP. We observed these problems in samples, which were positive for CMV and adenovirus. The importance of viral co-infections remains uncertain [11-13]. In summary, we detected multiple infections in 36% of the TS samples analyzed and the detection of multiple infections was only possible using the RespiFinder-19 assay. However, other studies also demonstrated the detection of multiple infections using the RVP assay [9,21]. In this context, the question arises, whether all detected viruses in multiple infections are clinically relevant or not. In some cases the pathogens of the co-infections cause more serious illnesses, e.g. coronavirus, RSV or hMPV, than the pathogens of the primary infection, e.g. rhinovirus. With this study approach, none of the three assays can differentiate between pathogens of the primary infection and the co-infecting virus. Therefore, the clinical relevance of additionally detected viruses has to be correlated in further studies.

The interpretation of the generated data of the three assays has different levels of difficulty. Abbott Molecular supplies with the RVP a software tool (TDAS RVP FAST software), which analyze the generated data and provided automatically a positive or negative signal for the detection of the particular virus. The interpretation of the results of the RespiFinder-19 is more difficult, because the manufacture did not provide helping data for the analysis (e.g. threshold). So it is possible that low positive samples were missed. The further developed RespiFinder-SMART-22, has an improved analysis of the generated data with given melting points.

In general, the comparison of the spectrum of pathogens detected in our patient cohort of mechanically ventilated patients with other studies (e.g. [14]) is hardly realizable, because the comparability is limited by regional, seasonal and methodological differences. In this context, the development of a respiratory networks (e.g. http://www.medical-dpc.com/respvir) will allow a constant comparison of seasonal accumulations and chronological trends.

For the application of the three multiplex PCR methods in a routine diagnostic setting, the hands-on-time, the time-to-result, the costs and the accomplishment are also very important parameters. Table 5 summarizes the specifications of RespiFinder-19, RespiFinder-SMART-22 and RVP. The time-to-result differs from three and a half hours with RVP to eight hours with RespiFinder-19. The RespiFinder-19 also needs more hands-on-time steps than the RVP. The RespiFinder-SMART-22, as a further development of the RespiFinder-19, delivers a result after six hours. This amounts to a time saving of one and a half hour. For a significant effect on disease progress, patients with respiratory tract infections need an efficient pathogen based prophylaxis or therapy. Multiplex PCR methods allow a fast and exact identification of the causative pathogens. For routine application the analytical sensitivity must balance out the time-to-result. In our opinion, the turn-around-times of the RespiFinder-SMART-22 and RVP assay did not have a significant impact on patient treatment, because both assays provided results within one working day. In this context, the RespiFinder-19 demands for a strict time management to achieve results within a day.

Study limitations: Unless samples were not analyzed in an adequate number of replicates to perform statistical analysis, our approach allow the comparison of the performance and the range of sensitivity of the three multiplex assays by analysis of inoculated samples with quantified virus material. Reported differences may be due to chance regarding a single consideration of individual results, however a tendency towards difference in lower or higher detection efficiencies by the different assays is observable.

## Conclusions

In conclusion, our study shows that before a multiplex PCR method is applied in routine diagnostics it has to balance out the analytical sensitivity between time-to-result, hands-on-time and the clinical relevance of the detected pathogens. The RespiFinder-19 has a higher analytical sensitivity than the RVP, but needs more than twice as long for a result compared to the RVP. The RespiFinder-SMART-22, as a further development provides a faster result, but has to been tested with routine samples in further studies.

## Competing interests

The authors declare that they have no competing interests.

## Authors' contributions

M.D.-R. conducted the experiments, analyzed the data and wrote the paper, T.V. coordinated the study, O.A. revising the manuscript critically for important intellectual content, C.K. and J.D. initiated the study, planned the experimental design and contributed to write the paper. All authors read and approved the final manuscript.

## Pre-publication history

The pre-publication history for this paper can be accessed here:

http://www.biomedcentral.com/1471-2334/12/163/prepub
